# SACTI model in prediction and assessment of large scale natural draft cooling tower environmental impact of nuclear power plant

**DOI:** 10.1038/s41598-023-38283-7

**Published:** 2023-07-10

**Authors:** Xuan Wang, Shuhuan Liu, Peng Cao, Jinsong Song, Chengkai Wang, Shanwei Xu, Shijie Zhu

**Affiliations:** 1 School of Energy and Power Engineering, Xi’an Jiaotong University, Xi’an, 710049 China; 2 Faculty of Architecture, Civil and Transportation Engineering, Beijing University of Technology, Beijing, 100081 China; 3 School of Civil Engineering, Dalian University of Technology, Dalian, 116024 China; 4 The 41st Institute of the Fourth Research Academy, China Aerospace Science and Technology Corporation, Xi’an, 710000 China

**Keywords:** Environmental sciences, Energy science and technology, Engineering

## Abstract

Large Scale Natural Draft Cooling Tower has become a hot topic in China because it is an important part of the nuclear power plant, and its environmental impacts include shading, solar energy loss, water deposition and salt deposition. In China, there is no built large-scale natural draft cooling tower of nuclear power plant. Therefore, model prediction becomes an effective way to solve this problem. This paper introduces the basic principles and structure of SACTI (Seasonal and Annual Cooling Tower Impact) model. SACTI is a cooling tower assessment model developed by Argonne National Laboratory, USA. A comparative case study between China's Pengze Nuclear Power Plant and the US Amos Power Plant is also presented. Calculations were carried out for the Pengze and Amos power plants, and the results showed that the maximum value of salt deposition at the Pengze plant was about 166.5 kg/(km^2^-month) at a distance of 800 m from the cooling tower. The maximum value of salt deposition at the Amos plant was about 92.85 kg/(km^2^-month) at a distance of 600 m from the cooling tower. Conclusions show that the research work can provide a useful solution in future work, the simulation results of the SACTI model have a potential mean in the absence of monitoring data. This research provides a way to generate simulation data through SACTI program in the design process of nuclear power plant cooling tower, and designers can use these data to determine how the cooling tower will affect the natural environment and manage within an appropriate range to reduce the impact on the environment.

## Introduction

With the continuous development of the national economy and the nuclear power industry, the construction of nuclear power plants has become an important planning goal in China. A large amount of steam is generated during the process of water and gas exchange in the large natural ventilation cooling tower of a nuclear power plant. Some of this steam can condense to form a white cloud, known as a "plume", when it is exhausted and mixed with the ambient air^1^. The particle size of the fog droplets in the plume is about 4–30 μm, due to the small particle size and light weight, it can reach a certain distance under the blowing of natural wind, and affect the solar radiation in a certain area, forming a large area of shadow, which is called "plume shadowing". This process will reduce the energy of solar radiation reaching the ground. In addition, when the nuclear plant's circulating cooling water splashes down in the cooling tower, it produces a large number of tiny droplets that are carried out of the tower by upward airflow, forming drift. Drift not only results in the loss of a large amount of circulating water. It also has an impact on the local environment. The circulating cooling water from the power plant cooling tower usually contains various types of impurities. These include soluble solids, suspended solids and other compounds. These substances will drift into the area around the cooling towers and cause salt deposits on the ground, which will affect the growth of local crops. The plume, shadowing, salt deposition and noise generated by the exhaust steam from the cooling tower will have some impact on the environment^2–6^. The quantitative analysis of these impacts has been a pressing issue in the environmental impact assessment of nuclear power plants. The environmental impact of cooling towers is also required to be assessed in the "Format and Content of Environmental Impact Report for Nuclear Power Plants" (NEPA-RG1) issued by the Ministry of Environmental Protection of the People's Republic of China.

In China, there are no completed nuclear power plants with large natural ventilation cooling towers. Therefore, it is impossible to obtain actual monitored data. Therefore, for large natural ventilation cooling towers in nuclear power plants, we can only use mathematical-physical models to estimate plume shadowing, drift and salt deposition. The rationale for siting nuclear power plants is directly affected by the reliability of the model results. On the other hand, the cooling towers of existing thermal power plants are relatively small in size. And the environmental impact studies of the cooling towers of domestic thermal power plants are mostly limited to the operating noise of the cooling towers. Cooling tower plumes have not yet been investigated and assessed.

A number of mathematical models of the environmental impact of cooling towers were proposed abroad in the 1970s. The ORFAD (Oak Ridge Fog and Drift) model^7,8^, developed by the Oak Ridge National Laboratory, can use real-time meteorological data to simulate the dispersion of a cooling tower plume. However, the ORFAD model is too time-consuming, and the model assumes that sunlight is always perpendicular to the ground, which does not accurately reflect the real situation. The KUMULUS model^9,10^, developed by the Swiss company Motor Columbus, is computationally optimized, and the model reflects the real situation by simulating several representative plume in the plant site. But the KUMULUS model always assumes that the plume can completely block the solar radiation during the calculation, thus the model results are too conservative. Argonne National Laboratory has developed a "second generation" cooling tower environmental impact model, SACTI (Seasonal and Annual Cooling Tower Impact), based on the existing first generation cooling tower environmental impact model. This model is based on the Cooling-Tower-Plume Prediction Code (CTPPC), which quantifies the length of the visible plume, drift, icing, shadowing of the plume, and the effects on solar radiation and salt deposition through single and multiple data inputs^11,12^.

Following the launch of the SACTI, the reliability of the system was tested under real-life conditions. Policastro et al.^13^ have used the KUMMULUS and SACTI models to simulate the extent and height of the visible plume for single and multiple cooling towers. The results show that for the extent and height of the visible plume, the SACTI model can match the data observed in the field.

Carhart et al.^14^, Carhart and Policastro^15^, and Policastro et al.^16^ used the SACTI model to predict the effects of natural ventilation cooling tower plume shading and to calculate the visible plume length, lift, and salinity. By comparing the calculated results with other previous models, they found that the SACTI model was better at handling meteorological data. Similarly, the SACTI model is better at predicting the length and height of the visible plume and salt deposition in cooling towers.

From November 1973 to March 1975, researchers from the United States used aerial measurements to observe the natural draft cooling towers at three thermal power plants. In total, more than 300 flights were made^17^. The conclusions of the measurements showed that the length of the visible fog plume did not only depend on the nature of the hot and humid air stream coming out of the cooling towers, but also on the relative humidity and the stability of the surrounding atmosphere. Uthe^18^ used LIDAR to measure the height of the cooling tower plume at the power station. He showed that the height of the plume measured by radar was about 10 times higher than that observed by the general method. However, the maximum height of cooling towers they observed did not exceed 150 m, while the height of cooling towers in nuclear power plants reached more than 200 m, and the operating conditions also differed significantly, so it is impossible to evaluate the environmental impact of cooling towers in nuclear power plants using the observed data from thermal power plants.

In this paper, we have used the SACTI programme for the prediction and calculation of the environmental impact of the cooling towers of the Jiangxi Pengze nuclear power plant. We have also compared and contrasted the results obtained with those obtained from foreign nuclear plants. The results show that the SACTI model can still be used for the analysis of the environmental impact of reactor cooling towers in China. Therefore, in the future design of cooling towers, it can be used to determine the environmental impact. Thus, the designer can reduce the environmental impact and avoid architectural violations by using the simulation data. This paper provides a relatively clear and reliable verification direction for the future construction and design of related facilities.

In this research, the influence of cooling tower wet deposition and salt deposition on the local environment under different cooling tower operating parameters is studied and given in combination with the operating characteristics of large natural ventilation cooling towers based on the SACTI model. A method for evaluating the influence of the cooling tower wet heat plume on the local environment is established.

## Overview of the SACTI model

### Introduction to the model

The SACTI model, which is known as Seasonal and Annual Cooling Tower Impact (SACTI)^19,20^, was developed by Argonne National Laboratory as a part of the EPRI (Electric Power Research Institute) project.

The basic principle of the model is the cooling tower plume prediction model. Factors influencing the diffusion of the cooling tower plume include meteorological elements, topography, nearby buildings and the height of the cooling tower^21–25^. In addition, ambient temperature and wind speed directly affect the water vapour height and the diffusion range of the plume. Therefore, under different conditions of ambient temperature and wind speed, the SACTI model can simulate the variation of the characteristic parameters of the plume. In this way, the SACTI model can be used to analyse how the plume will react to different ambient temperatures and wind speeds and what the possible effects will be^26–28^.

The SACTI model can now calculate the environmental impact of a single tower as well as multiple towers lined up in a given direction. The results include the length-frequency distribution of the plume in a subzone of 16 directional and distance segments around the cooling tower, and the distribution of cumulative solar radiation loss, water deposition and salt deposition over the year.

The SACTI model includes humidity and temperature as data inputs, and the current effects of humidity and temperature on plume dispersion are reflected in the fact that as temperature increases and humidity decreases, water droplets in the plume will evaporate faster, and these effects are considered in the SACTI model.

In order to verify the accuracy of the model, Carhart^29,30^ used 39 sets of observed data from single towers and 26 sets of observed data from multiple towers to compare the simulated data from SACTI for the study. In 60% of the cases, SACTI was found to be able to control the error factor to within 2. For the shadow effect, the SACTI model takes into account the temporal shape, direction and optical thickness of the fog plume. Thus, it can reflect the radiation loss caused by the fog plume more realistically than previous models.

It is worth noting that SACTI tends to overestimate the fog plume length, and this bi-conservative result is useful in environmental impact assessment.

### Basic structure of the model and input parameters

The SACTI model consists of four relatively independent subroutines to implement the model calculation. These are the pre-processing module (PREP), the plume calculation module (MULT), the table output module (TABLE) and the plotting module (PAGEPLOT), and the four modules are run sequentially.

**Figure 1:**
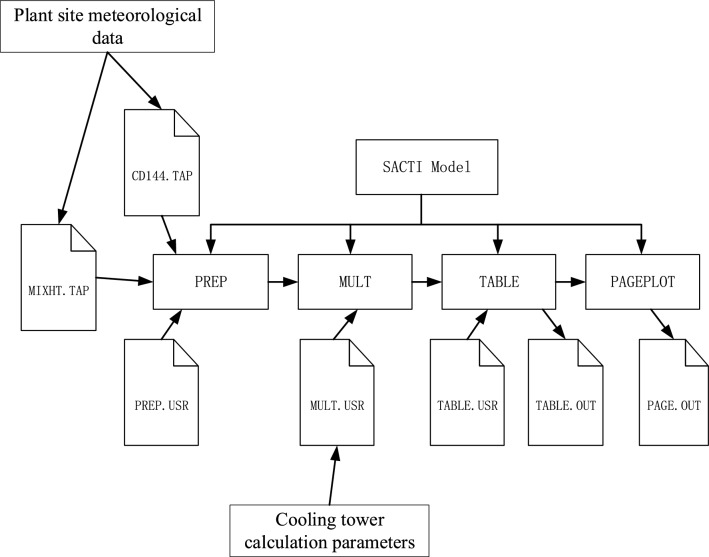
Structure of SACTI model.

The main structure of the SACTI model is shown in Fig. 1.

The pre-processing (PREP) module mainly filters the input data and removes the invalid and incorrect data to generate typical conditions for cooling tower outlet section conditions, with a total of 35 conditions generated in the SACTI model. The pre-processor requires three types of data input from the user: hour-by-hour meteorological data (CD144.TAP), day-by-day mixed layer height data (MIXHT.TAP), and source term data (PREP.USR). The plume calculation (MULT) module calculates the impact range of plume and drift using typical operating conditions for each type of plume. The input file (MULT.USR) for this module is relatively simple and allows the user to customize the number of cooling towers and the relative coordinates of each cooling tower. The TABLE module use an enhanced database and plume calculation program to generate the final results and produce a text data table that reflects the environmental impact at different wind directions and different seasons. The input control file of the module is TABLES.USR, which can be controlled by the user according to the needs of output. For example, when the user wants to output the distance segment interval or vertical height segment interval, the output results of the module are saved in the Table.OUT file. PAGEPLOT program will print out the results in the table program, the format of the output is ASCII graphics file PAGE.OUT.

### Validation of the SACTI model

This paper uses experimental data from Chalkpoint Power Station^29^ to verify the validity of the SACTI model. Although extensive field tracer tests of salt deposition have been carried out at Chalkpoint, the results had often been unsatisfactory due to the influence of other nearby stack emissions. The most favourable test conditions were achieved on 16 June 1977. The results of this tracer test are used in this research for the model validation analysis.

The ChalkPoint plant tracer experiment used a single dose of 30 gallons of 20% tracer into the drench tank at the base of the cooling tower with no water replenishment or drainage, so the only means of moisture loss was through the cooling tower mist plume. The concentration of tracer in the drench tank was kept constant throughout the experiment and the plant operating load remained stable throughout the experiment. The source measurement report shows that the cooling tower plume caused a moisture loss of 0.002%. The plume temperature was 315.3 K, the ambient temperature was 295.3 K, the plume discharge rate was 4.5 m/s, the tracer discharge rate was 1.86 g/s, the ambient humidity during the test was as high as 93%, so the evaporation of water droplets in the plume could be ignored, the wind direction during the test was south, so the cooling tower plume diffusion basically did not receive the influence of other plant site structures, the wind speed during the test period at a height of 100 m is about 8 m/s, below 100 m, the wind speed approximately obeys the exponential distribution, the wind speed at a height of 50 m is about 5 m/s. The monitoring point layout to the cooling tower as the central point, the distance from the cooling tower 0.5 km and 1.0 km set 35° arc, every 5° set a sampling point.

**Figure 2:**
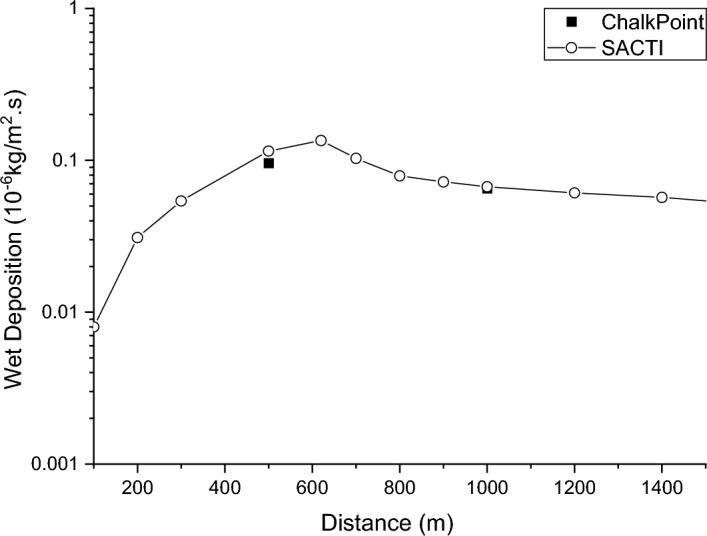
Comparison of water deposition concentrations at ground level in the ChalkPoint power plant.

The surface wet deposition concentrations at these two distances were used for the validation analysis, as the most favourable results from the ChalkPoint experiment were obtained for the 500 m and 1000 m arc sampling data. Figure 2 shows a comparison of the SACTI results with the experimental results.

The SACTI calculations are slightly higher than the ChalkPoint experiment results at 500 m and in better agreement at 1000 m, as shown in Fig. 2. The maximum wet deposition concentration at ground level with a wet deposition of 1.35E−07 kg/m^2^ s occurs at 620 m from the SACTI calculations.

The ChalkPoint power plant experiment is the most successful experiment for wet plume deposition in large naturally ventilated cooling towers. For wet deposition sampling, the ChalkPoint power plant experiment has two arc sampling points and this validation has been carried out using data from the largest monitoring point on the two arcs, and there are two experimental data points in Fig. 2. This validation process is more similar to that carried out by Meroney^31^. which also used 2-point data to validate. Again, the calculated results of the SACTI model are in good agreement with the experimental results, as shown by the validation results of this paper.

### Application and analysis of SACTI model

This paper applies the SACTI model to predict the environmental impact of two large natural ventilation cooling towers of the Jiangxi Pengze nuclear power plant, and the simulation results of the SACTI model at the Amos power plant in Chicago, USA, are analogously analyzed with the prediction results of this paper.

### Meteorological data

Due to the relatively long operation of the Amos power plant, there is no longer access to the latest meteorological data, and only meteorological data from 1981 are available for reference. The observation data of Pengze power plant are only publicly available for 2009, so these meteorological data are used in this study.

**Figure 3:**
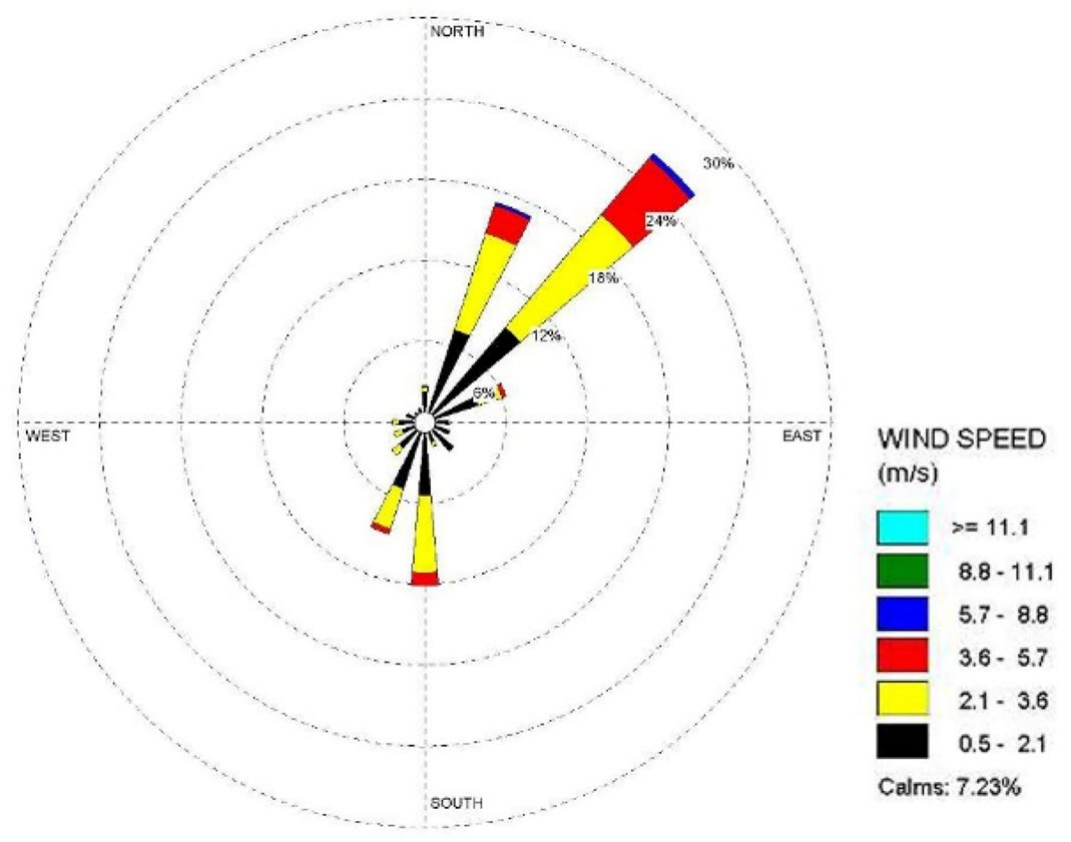
2009 Annual wind rose of Pengze nuclear power plant.

Jiangxi Pengze nuclear power plant site is located in the south of China, the average relative humidity of the area where the plant is located in 2009 is 78.5%, the average annual wind speed is 3.3 m/s, the dominant wind direction is NE wind direction throughout the year, Fig. 3 shows the annual wind rose map of the area where Jiangxi nuclear power plant site is located, this paper uses the meteorological data observed by local weather stations. The Pengze nuclear power plant site is located in the Jiangxi province of China and is one of the first nuclear power plant sites to be built in China. After the Fukushima nuclear accident, the construction of the Pengze site was stopped due to the change in Chinese policy. Therefore, the latest meteorological data of the Pengze site is only available for 2009. In addition, this paper mainly describes and discusses the functions of the SACTI model. It does not analyse the effectiveness of specific projects. Therefore, as long as the meteorological data of the plant site can guarantee 8760 h of data to meet the computational requirements of the SACTI model, the analysis results presented in this paper are still reasonable and credible.

**Figure 4:**
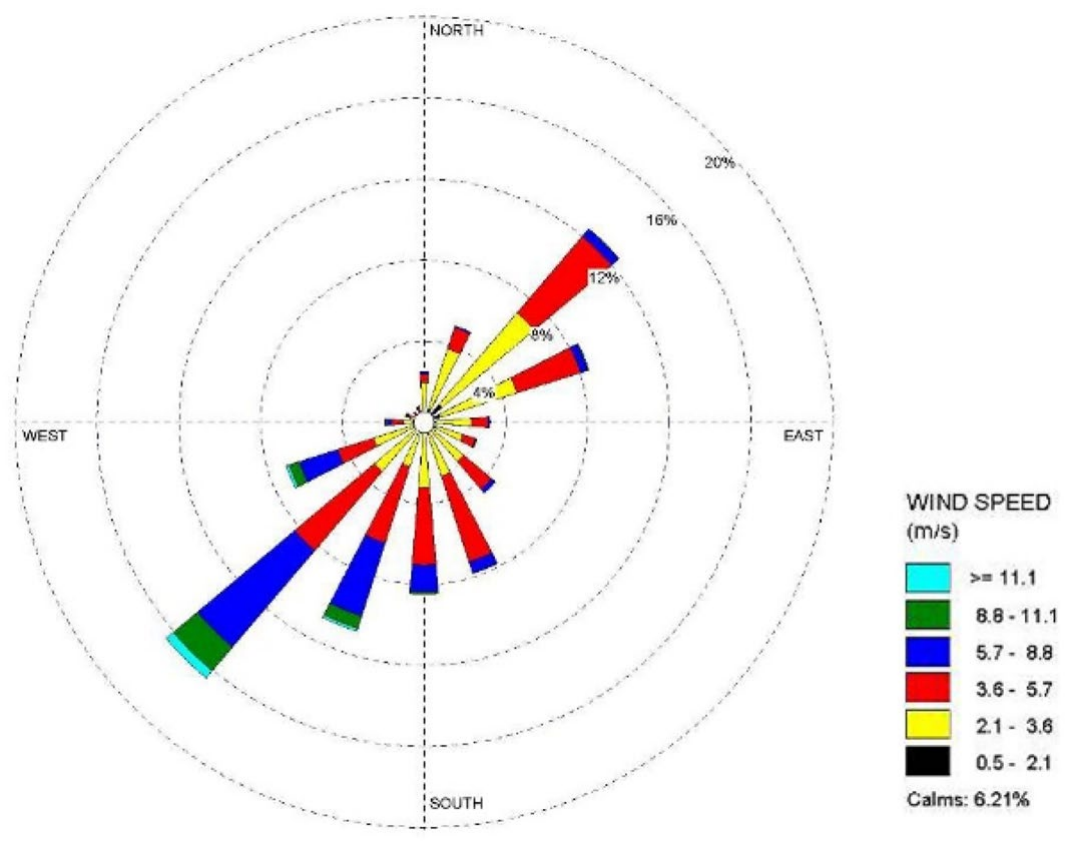
1981 Annual wind rose of Amos power plant.

Figure 4 shows the wind rose map of the whole year of 1981 in the area where Amos Power Plant is located. The annual dominant wind direction is SW wind direction, the annual average wind speed is 7.4 m/s, and the annual average relative humidity is 70.8%.

### Main parameters of cooling tower

During the calculation, the geometric center points of two natural draft cooling towers of Pengze Nuclear Power Plant and Amos Power Plant are both used as the origin coordinates of the model input. According to the cooling tower process design of Jiangxi Pengze Nuclear Power Plant, the main cooling tower calculation parameters are shown in Table 1 during SACTI calculation.

**Table 1:** Main designing parameters of cooling tower.

Parameters	Pengze nuclear power plant	Amos power plant
Thermal load	2168 MW	1216 MW
Height	215 m	124 m
Effective diameter of cooling tower outlet	102 m	113.76 m
The entry speed of cold air at the bottom inlet of the cooling tower	49,833 kg/s	55,000 kg/s
Cooling tower type	Natural ventilation cooling tower	Natural ventilation cooling tower
Fill type	Splash fill	Splash fill
Fill height	13.3 m	8.6 m
Drift eliminator	High efficiency water collector	General water collector
Entrance height	12.4 m	8 m

## Simulation results

**Figure 5:**
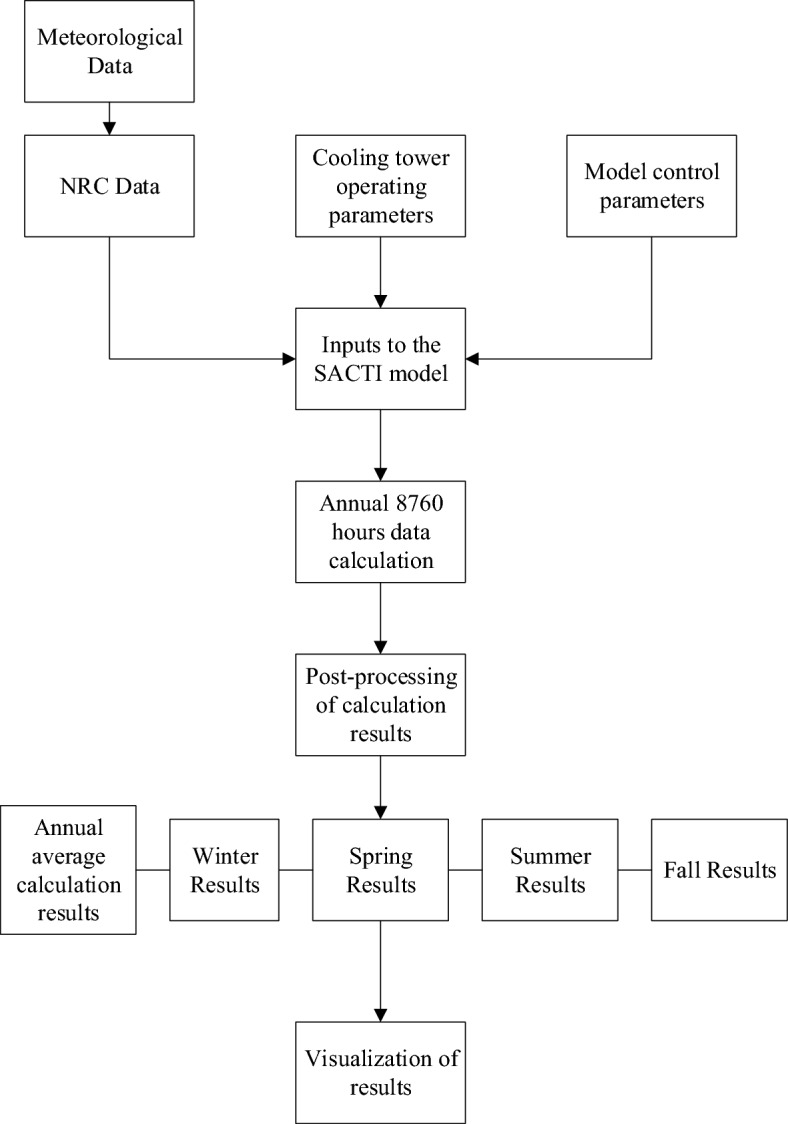
Simulation flow chart.

The simulation flow chart of this paper is as shown in Fig. 5.

### Distribution of plume and plume shadowing

**Figure 6:**
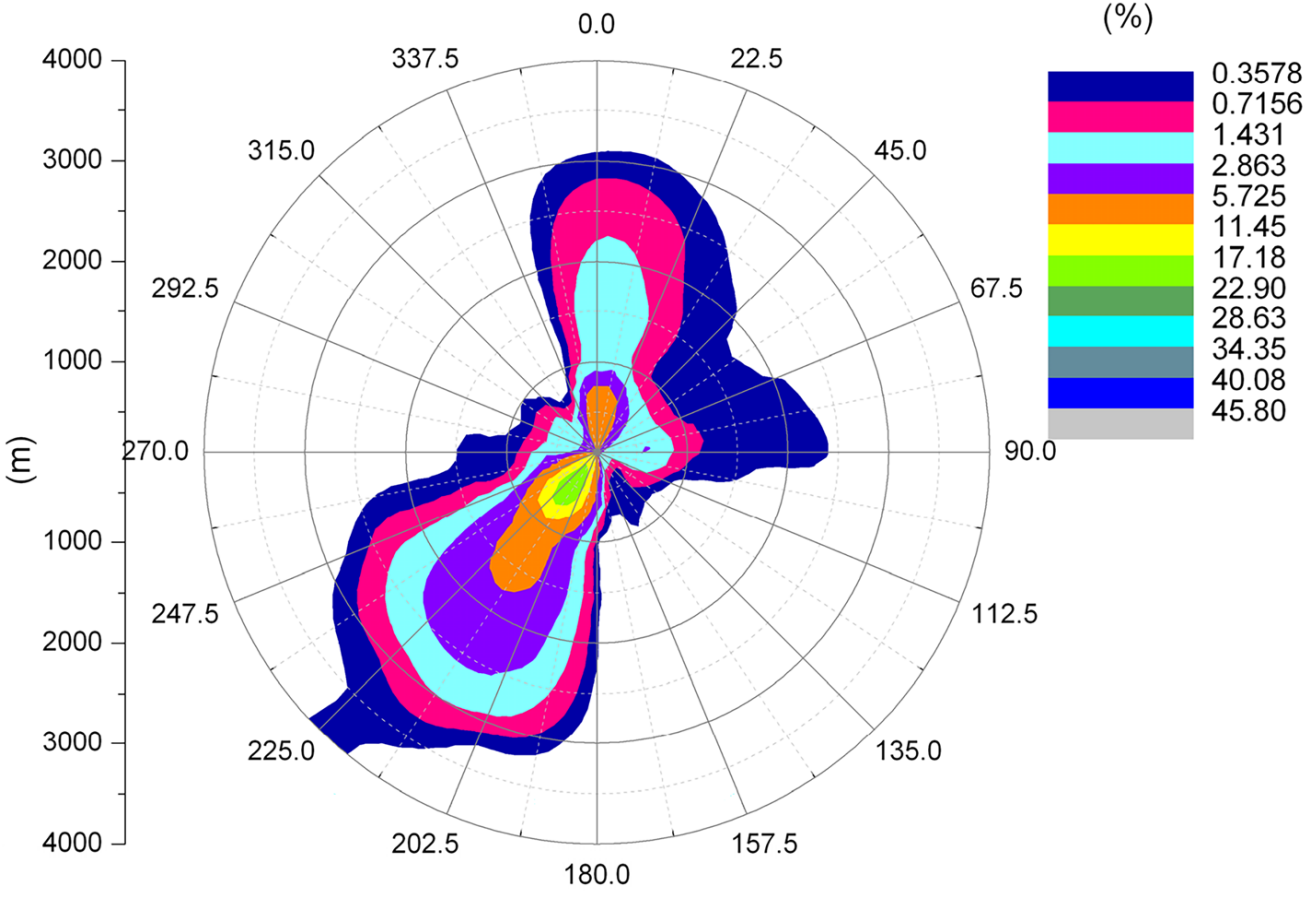
Annual plume length frequency of Pengze nuclear power plant.

**Figure 7:**
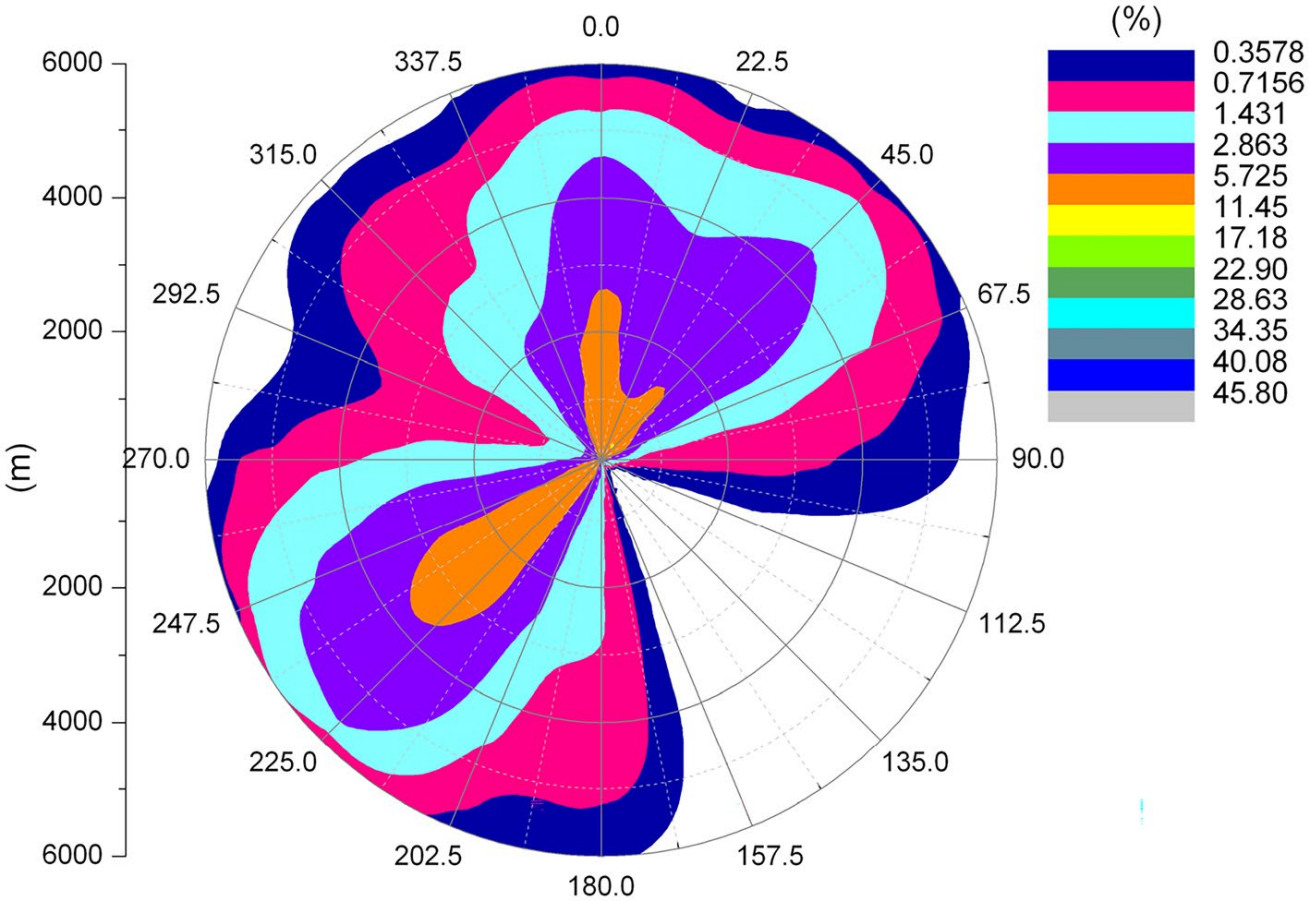
Annual plume length frequency of Amos power plant.

**Figure 8:**
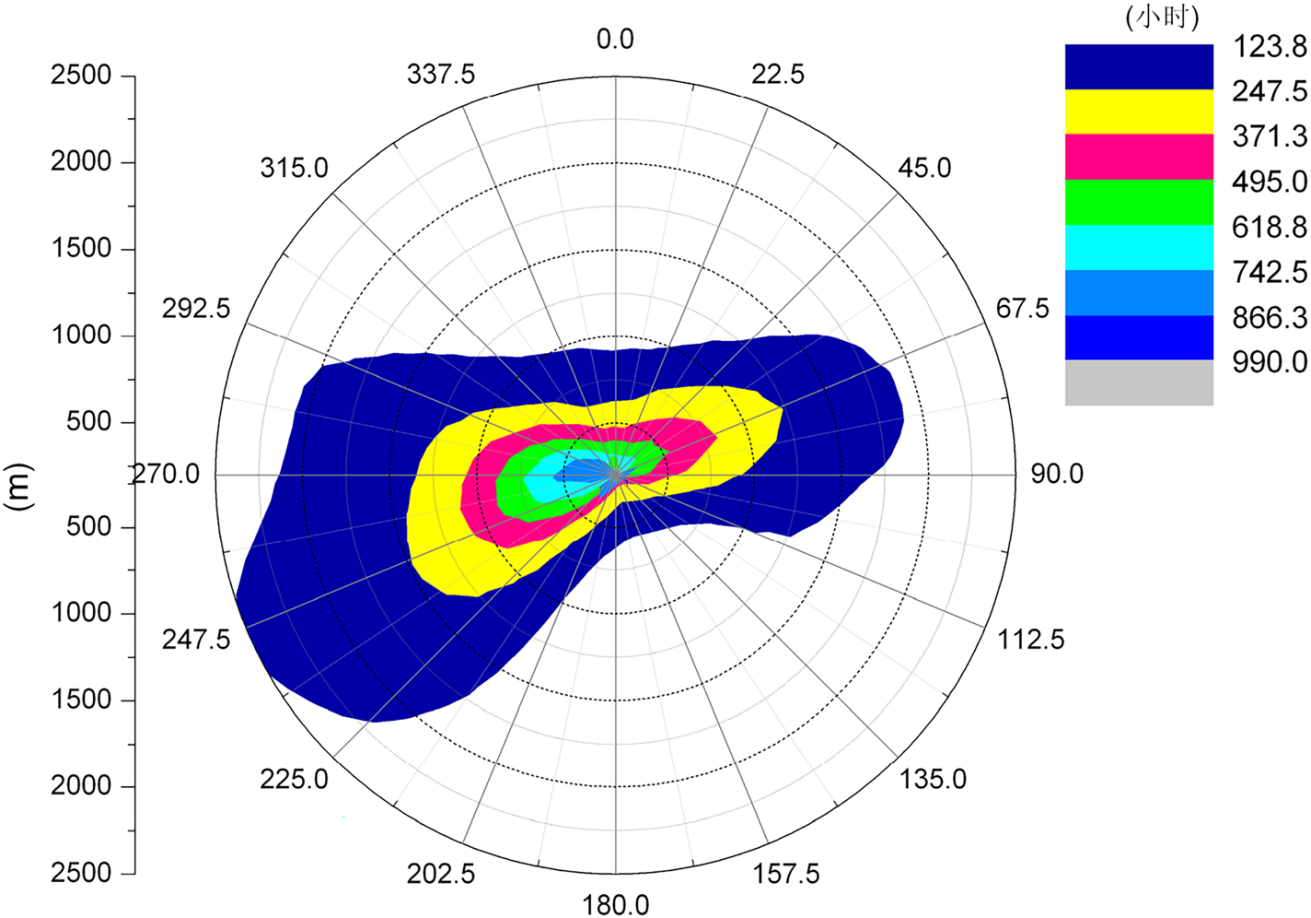
Annual shadowing hours of Pengze nuclear power plant.

**Figure 9:**
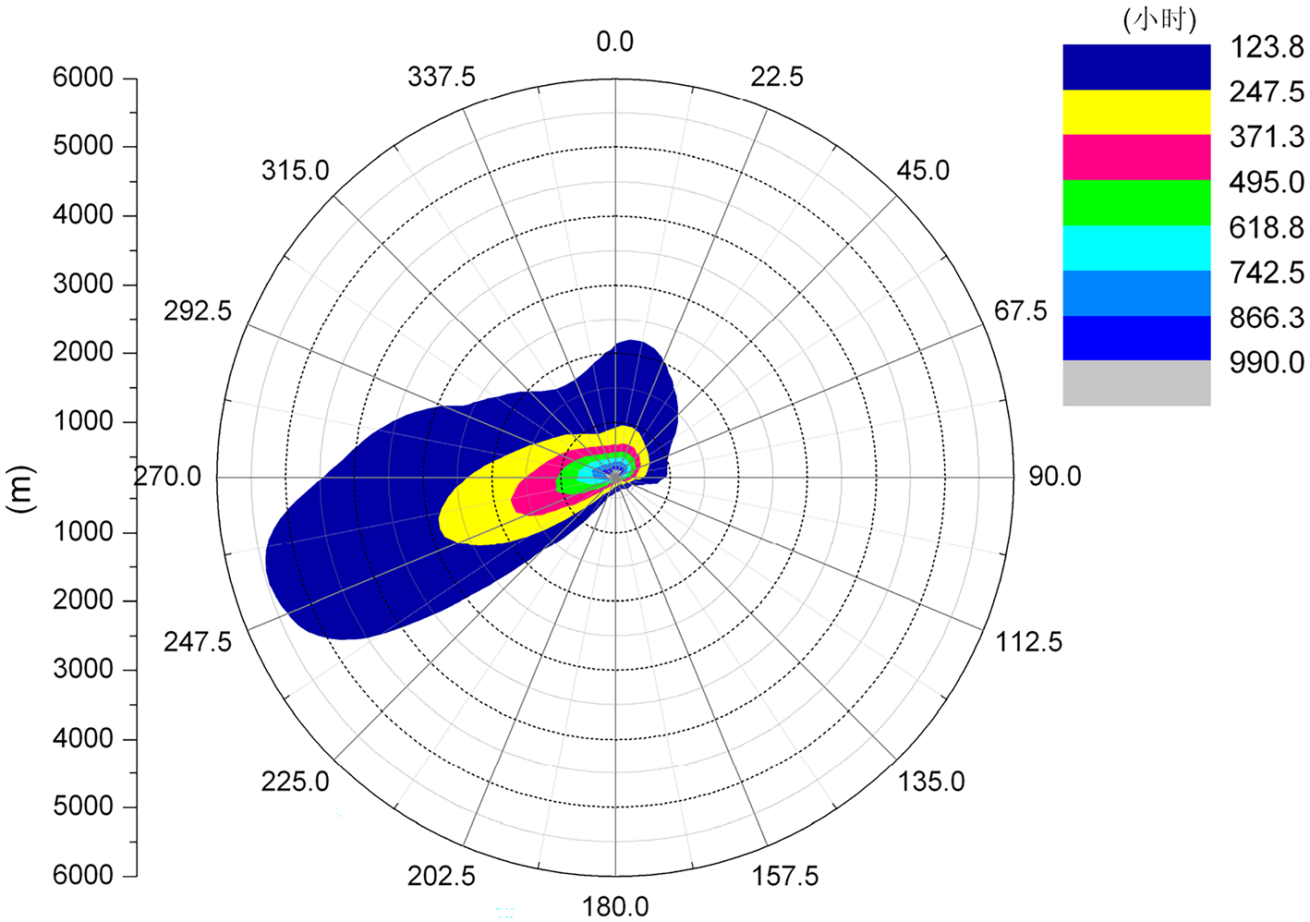
Annual shadowing hours of Amos power plant.

**Figure 10:**
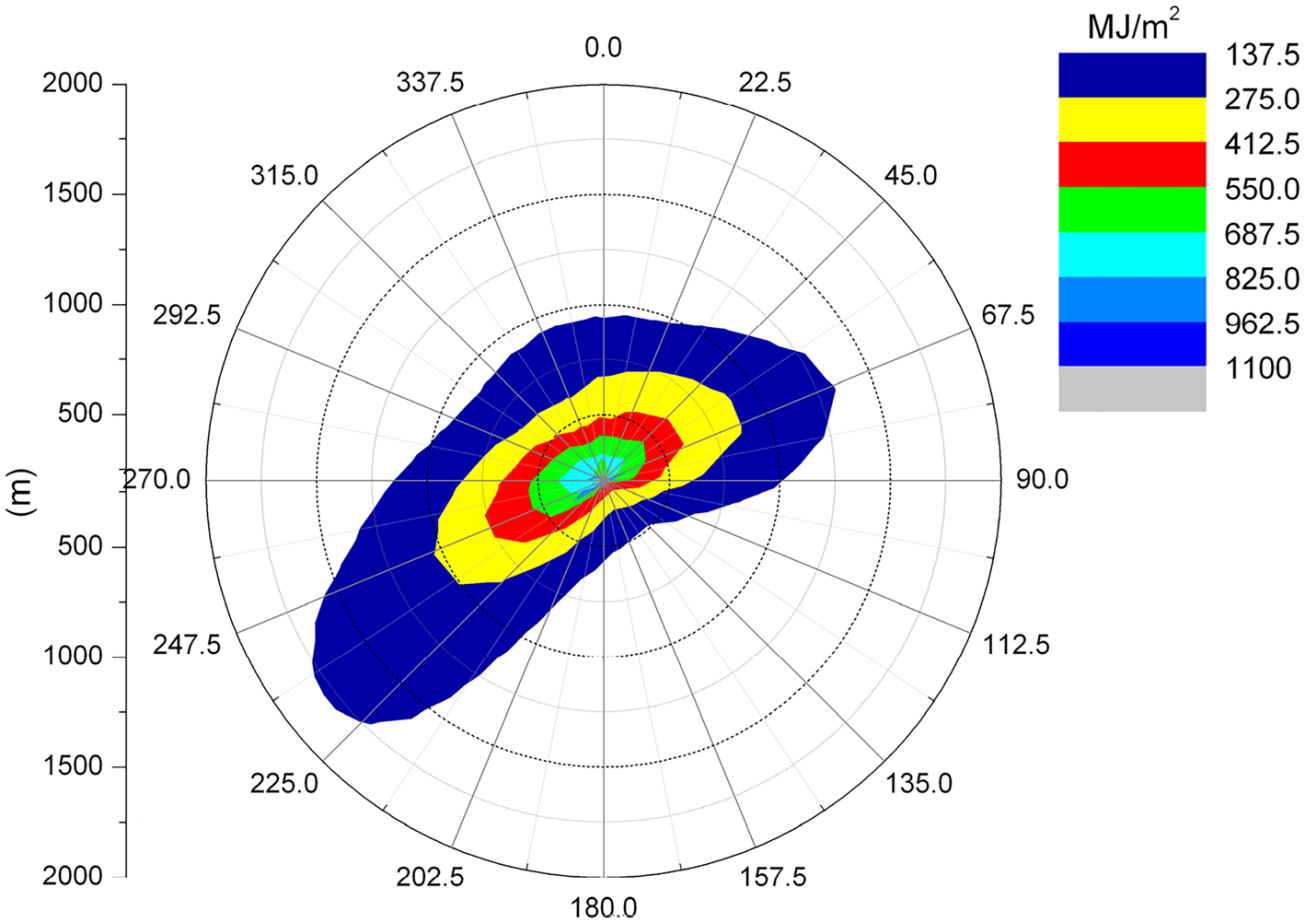
Annual solar energy loss of Pengze nuclear power plant.

**Figure 11:**
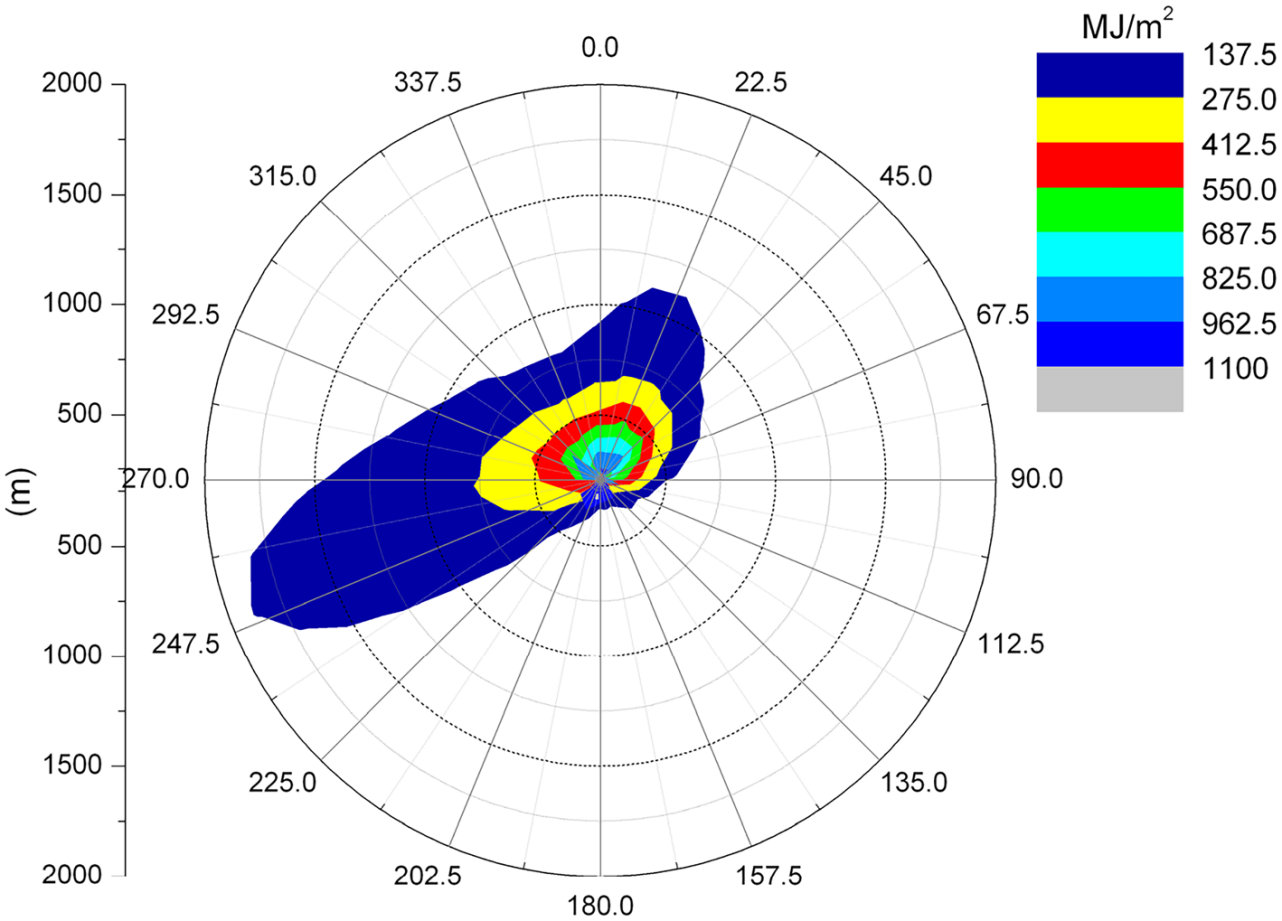
Annual solar energy loss of Amos power plant.

Figures 6 and 7 show the length frequency distribution of plume of cooling towers in each direction for Pengze Nuclear Power Plant and Amos Power Plant, Figs. 8 and 9 show the number of hours of “shading” caused by plume around cooling towers for Pengze Nuclear Power Plant and Amos Power Plant throughout the year, and Figs. 10 and 11 show the distribution of accumulated solar radiation loss around cooling towers for the two power plant. Figures 10 and 11 show the distribution of cumulative solar radiation loss at various distances and within the region throughout the year.

Figure 6 shows that the cooling tower plume length frequency distribution of the Pengze nuclear power plant mainly occurs at the geometric center of the cooling tower in the SW and N directions, which is basically consistent with the wind frequency distribution in the wind rose diagram shown in Fig. 3 i.e., the area with the largest cooling tower plume length frequency occurs in the downwind direction of the dominant wind direction throughout the year at the site, mainly concentrated in the range of 3000 m from the cooling tower. The cooling tower plume length frequency distribution of Amos power plant shown in Fig. 7 has a similar pattern to that of Pengze power plant, which is consistent with the wind frequency distribution shown in Fig. 4, but the distance range of the cooling tower plume length frequency distribution of Amos power plant is about 6000 m, which is twice as far as that of Pengze nuclear power plant. When comparing the annual wind speed of the two sites, we can find that the annual average wind speed of Amos power plant is 7.4 m/s, while the average annual wind speed of the Pengze nuclear power plant is only 3.3 m/s, indicating that the length of the cooling tower plume is largely influenced by the wind speed.

From Fig. 8, we can find out that the plume shadowing phenomenon caused by the cooling tower plume of Pengze NPP mainly occurs in the WSW and ENE directions, and from Fig. 8, the plume shadowing of Amos NPP mainly occurs in the WSW and NNE directions, which may be related to the relative humidity of the atmosphere at the site of the nuclear power plant. The percentage of solar radiation loss shown in Figs. 10 and 11 is basically consistent with the distribution of plume shadowing, and also shows that the plume shadowing phenomenon is the main cause of solar radiation loss. The maximum solar radiation loss caused by the cooling tower plume of both Pengze Nuclear Power Plant and Amos Power Plant occurs at a distance of 200 m from the cooling tower, and the corresponding reduction in solar radiation energy is 1005 MJ/m^2^ and 1231.5 MJ/m^2^, accounting for about 2.78% and 3.94% of the total solar radiation energy. The natural interannual fluctuation range of solar radiation is about 1% to 10%. The shading caused by the cooling tower plume during normal operation of the nuclear power plant is mainly concentrated in a limited area around the plant site, and the maximum solar radiation energy loss caused by shading is only 2.78% and 3.94% of the solar radiation, which is within the natural inter-annual fluctuation range of solar radiation. Therefore, it is expected that the formation of “plume shadowing” from the cooling towers plume will generally have no significant impact on the surrounding environment and terrestrial ecology.

### Droplet and salt deposition

The measured droplet spectra of Chalk Point Power Plant in the United States^29,30^ showed that the droplet diameters of naturally ventilated cooling towers are in the range of about 10–2000 μm, mainly concentrated in the diameter range of 10–70 μm, accounting for about 56% of the total mass.

**Figure 12:**
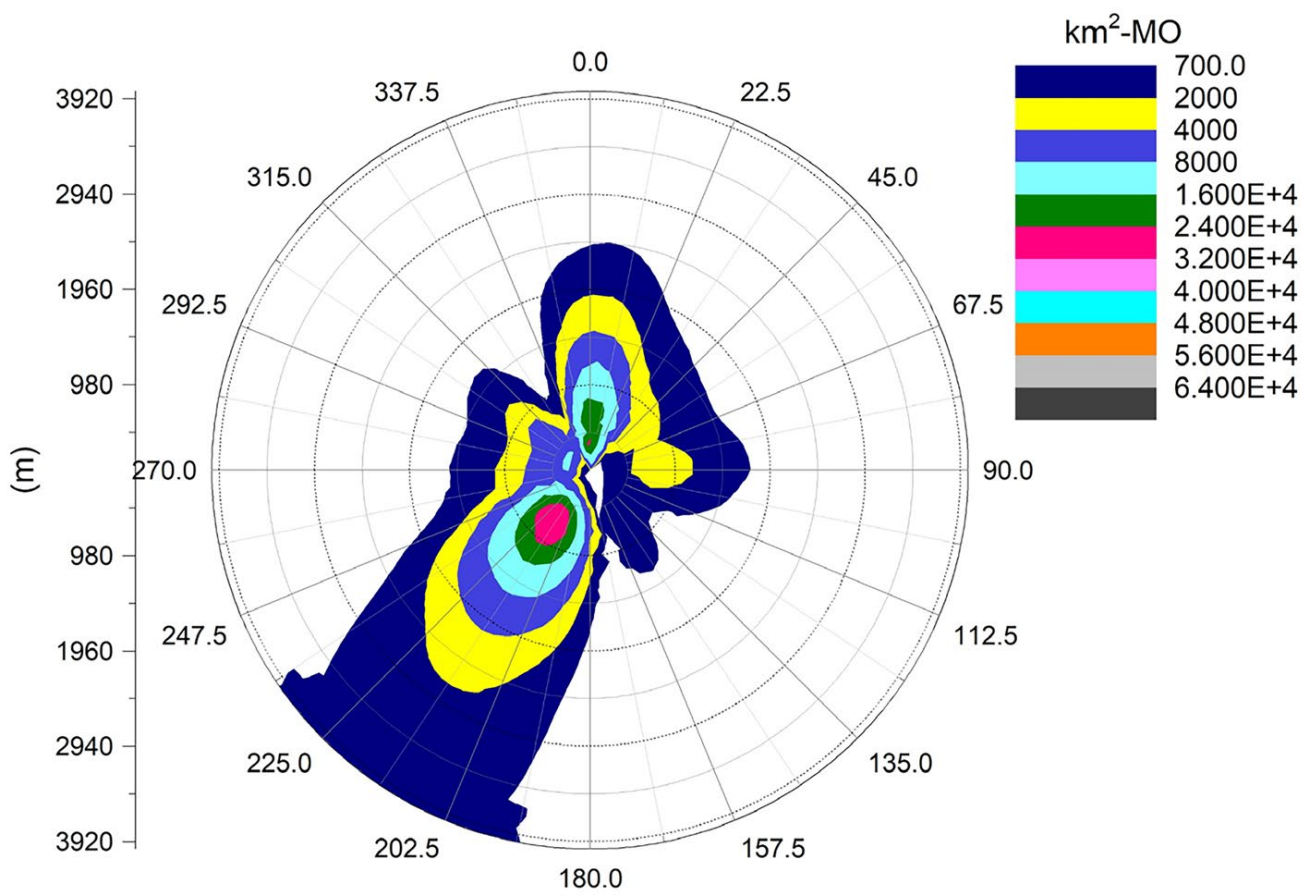
Annual water deposition of Pengze nuclear power plant.

**Figure 13:**
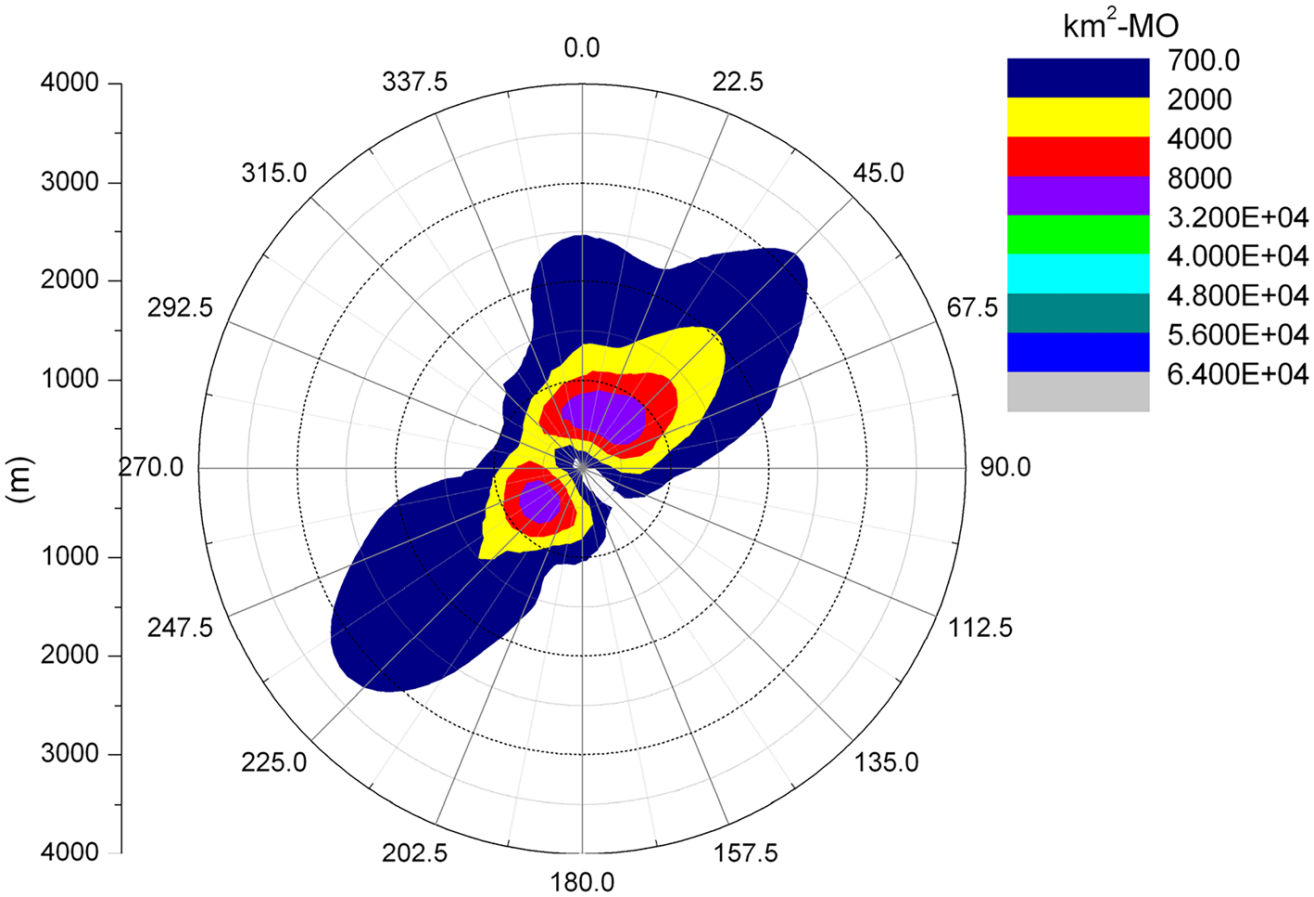
Annual water deposition of Amos power plant.

It can be seen from Fig. 12 that the ground deposition water caused by the cooling tower plume at the Pengze nuclear power plant is mainly distributed in the SW and N directions at the geometric center of the cooling tower, which is consistent with the length frequency distribution of the plume in Fig. 5, and both directions are downwind of the main wind direction of the site throughout the year, which also indicates that the main factor affecting the distribution of ground deposition water is the wind frequency. Figure 13 shows the distribution of ground-deposited water caused by the cooling tower plume of Amos power plant, which is basically consistent with the plume length frequency distribution shown in Fig. 7. The maximum value of ground-deposited water at the Pengze nuclear power plant occurs at 800 m from the cooling tower, which is about 3.0E+04 kg/(km^2^·month), equivalent to an increase in precipitation of 0.4 mm/year, while the maximum value of ground deposited water at the Amos power plant occurs at 600 m from the cooling tower, which is about 1.50E+04 kg/(km^2^·month), equivalent to an increase in precipitation of 0.18 mm/year. Comparing the design parameters of the two cooling towers listed in Table 1, it can be found that the thermal load of the cooling towers of the Pengze nuclear power plant is about 1.8 times that of the cooling towers of the Amos power plant, and the height of the cooling towers is about 1.7 times, thus the difference in the amount of deposited water caused by the plume of the cooling towers of the two power plants may be related to the thermal load and height of the cooling towers. At the same time, the average annual precipitation in the area of Pengze nuclear power plant site is about 1346.6 mm, and the maximum precipitation caused by cooling tower drift predicted by the SACTI model is much lower than the natural precipitation. The maximum percentage of ground solar loss of Pengze nuclear power plant occurs at 200 m, while the maximum value of ground deposition water occurs at 800 m and 600 m respectively, indicating that the distribution of deposition water lags behind the ground solar loss, which is mainly due to the fact that the cooling tower plume is generally distributed in a relatively high range, and the condensation water continues to drift downwind under the influence of wind.

CFD computational fluid dynamics software is also currently available to carry out environmental impact assessments of cooling tower wet heat plumes.

One key difference between CFD and the SACTI model is that CFD is much more computationally-intensive and requires more detailed input data, whereas the SACTI model can be run relatively quickly and with less detailed input information. Additionally, CFD can provide a much more detailed understanding of the fluid dynamics and pollutant dispersion within the cooling tower and its surrounding environment, while the SACTI model is limited in its ability to capture these complexities. However, the SACTI model is a useful tool for quickly assessing the environmental impact of cooling towers in a nuclear power plant setting, and its simplicity makes it accessible to non-experts in fluid dynamics and computational modeling.

In previous research, we use CFD software Star-CCM+ to study the wet deposition of wet heat plumes in cooling towers, with the following analysis process:

The results of the CFD and SACTI models were analysed for the axial concentration of wet deposition. In the immediate area, the CFD and SACTI results were relatively close, but beyond 2 km, the SACTI results for wet deposition on the ground decreased more significantly with increasing distance. At 1 km, the CFD results are slightly higher than the SACTI results, mainly because the CFD calculations take into account the effect of the large naturally ventilated cooling tower structures themselves on the dispersion of the plume.

The maximum value of CFD ground wet deposition is 2.48E−08 kg/m^2^ s, which occurs 300 m downwind of the cooling tower, while the maximum value of SACTI simulated ground wet deposition is 9.65E−09 kg/m^2^ s, which occurs 900 m downwind of the cooling tower. The CFD simulation results are significantly more advanced than SACTI, indicating that after taking into account the influence of the cooling tower structures, the large value of ground wet deposition occurs significantly earlier. large values of ground-level wet deposition occur at significantly earlier distances after accounting for the effect of the cooling tower structures.

### Salt deposition

The following figure shows the average annual salt deposition distribution for the Pengze and Amos nuclear power plants.

**Figure 14:**
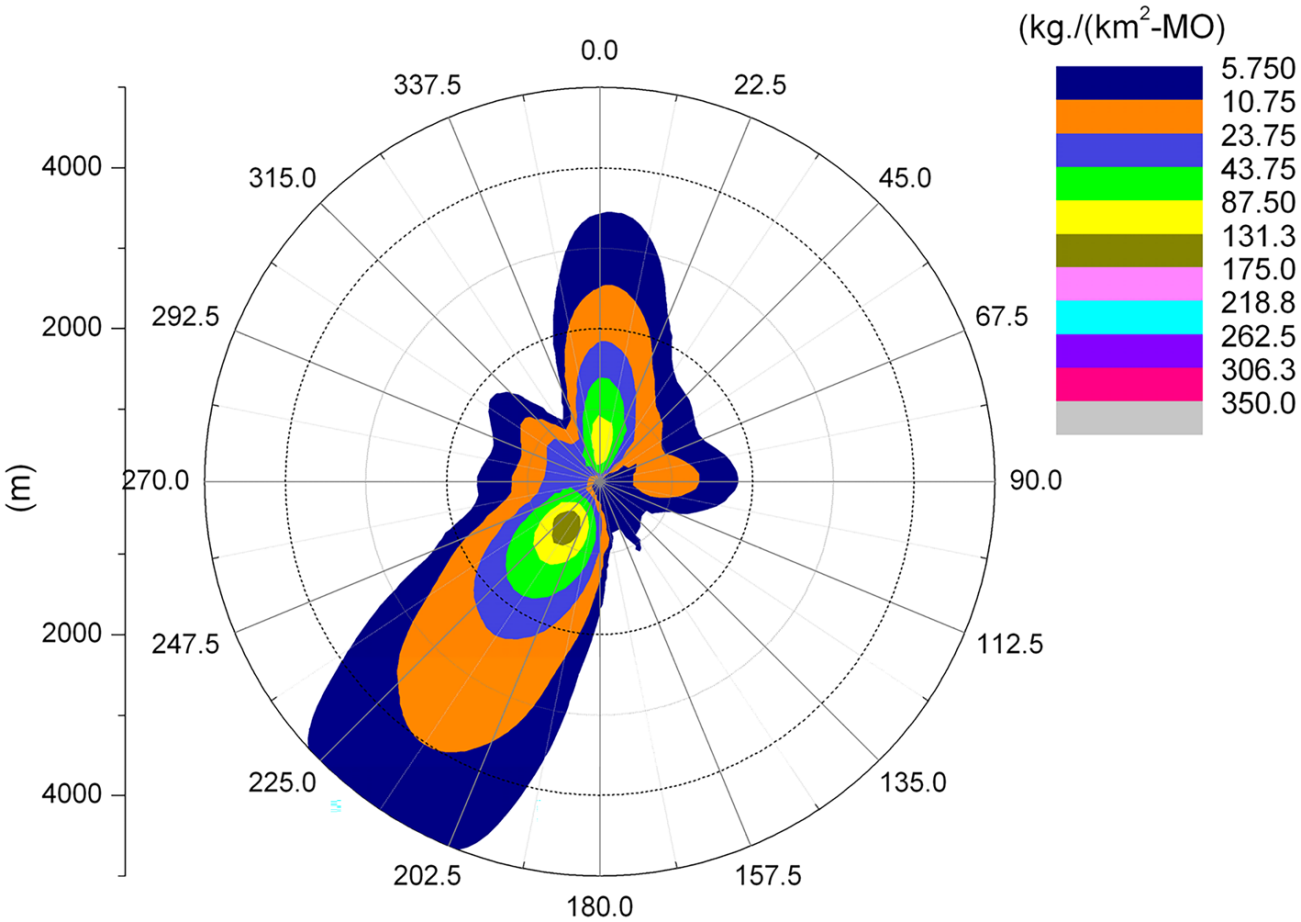
Annual salt deposition of Pengze nuclear power plant.

**Figure 15:**
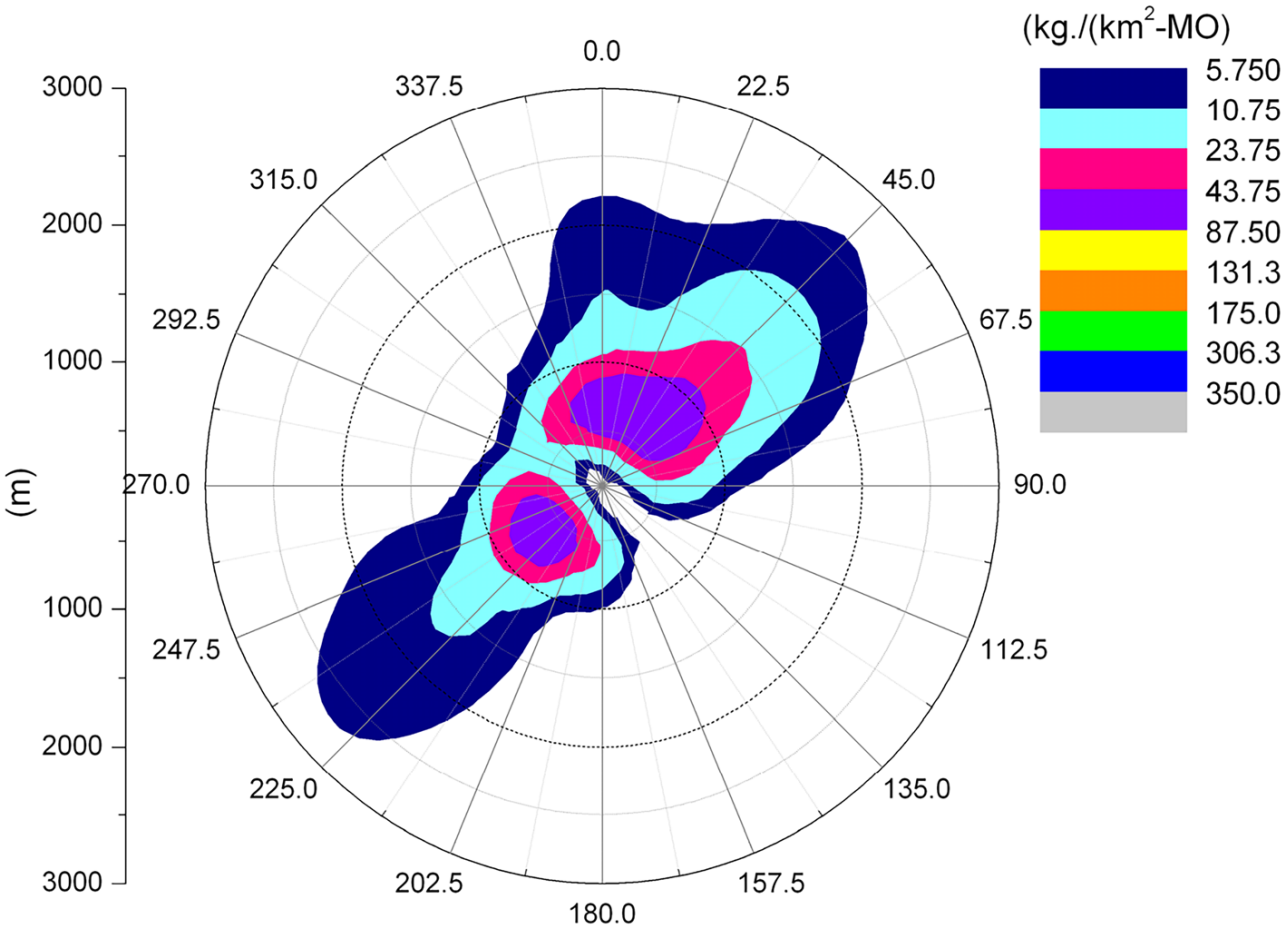
Annual salt deposition of Amos power plant.

From Fig. 14, it can be seen that the salt deposition in the Pengze nuclear power plant is mainly distributed in the SW and N directions of the geometric center point of the cooling tower, which is consistent with the surface water deposition shown in Fig. 10. Comparing Figs. 13 and 15 and, the Amos power plant also shows a similar regularity, with the maximum value of salt deposition in the Pengze nuclear power plant occurring at a distance of 800 m from the cooling tower, which is about 166.5 kg/(km^2^·month), the maximum value of salt deposition at the Amos plant occurs at a distance of 600 m from the cooling tower and is about 92.85 kg/(km^2^·month). Comparing the salt deposition data of the two plants, it can be found that the regularity of salt deposition and surface water deposition maintain a high degree of consistency. The guidelines for evaluating the effects of salt deposition rates on plants in NUREG-1555 of the U.S. Nuclear Regulatory Commission give: when the salt deposition rate in drift is 1–2 kg/(ha·month), there is no harm to plants; when the salt deposition rate approaches or exceeds 10 kg/(ha· month) during the growth period of plants (equivalent to 1000 kg/km^2^·month), it may cause harm to the leaves of many plants. The maximum salt deposition rate simulated by the SACTI model for the Pengze NPP is 166.5 kg/(km^2^·month), and therefore no significant effects on surrounding crops and other plants can be expected.

## Conclusion and prospect

In this research, we use SACTI model to predict the cooling tower plume for the Pengze nuclear power plant in Jiangxi, China and the Amos inland power plant in the United States. The following conclusions have been reached.

1. The main influence on the length-frequency distribution of the cooling tower plume is the wind speed and direction around the plant site. The average wind speed in the area of the plant site is 3.3 m/s for the Pengze nuclear power plant in China. The concentration of the plume is mainly in the area of 3000 m from the cooling tower.
2. There is a certain deviation between the time distribution of the plume shadow caused by the cooling tower plume and the wind frequency rose diagram of the area where the plant is located. This may be related to the relative humidity of the atmosphere in the area where the nuclear power plant is located. Simultaneously, the distribution of solar radiation loss caused by the cooling tower plume and the distribution of shading time are consistent, indicating that the shading phenomenon is the main cause of solar radiation loss. The maximum value of the solar radiation loss caused by the cooling tower plume occurs at a distance of 200 m from the cooling tower in both of the plants.
3. Cooling tower plume caused by the distribution of ground settling water and plume length frequency distribution basically remains constant, ground settling water can be related to the cooling tower heat load and height, while the distribution of ground settling water to lag behind the ground solar radiation loss, which is mainly due to the cooling tower plume is generally distributed at a relatively high height, condensation water continues to drift downwind under the influence of wind. The results showed that the maximum value of salt deposition in the Pengze plant was about 166.5 kg/(km^2^-month) at a distance of 800 m from the cooling tower, and the maximum value of salt deposition in the Amos plant was about 92.85 kg/(km^2^-month) at a distance of 600 m from the cooling tower. The regularity of the amount of salt deposition caused by the cooling tower plume maintains a high degree of consistency with groundwater deposition. Also, according to the predicted results of this paper for the Pengze nuclear power plant, the salt deposition from the large naturally ventilated cooling tower plume of the nuclear power plant will not have a significant impact on the surrounding crops and other plants. In addition, when the annual average wind speed of the plant site is small, the calculation results of the SACTI model will increase significantly, and it will be more obvious in the vicinity of the plant site. This is mainly because the calculation results of the Gaussian model are generally large under the calm wind conditions.
  Of course, in the SACTI model calculation, the water particle size distribution in the wet heat plume will also significantly affect the wet deposition distribution near the plant site. In general, below 300 microns, the water particles will produce an obvious evaporation process, causing the wet deposition on the ground to become less, while when the particle size is larger than 500 microns, the water particles will rapidly fall to the ground.
4. The SACTI model allows the quantitative calculation and simulation of the effects on the environment of cloud shadows, water and saline discharges during normal operation of large natural ventilation cooling towers in nuclear power plants, based on meteorology data over time. Although the SACTI model does not incorporate topographic correction and computational components, and the results of the simulations can be inaccurate, the results of the model calculations can be used as a basis for assessing the environmental impact of nuclear power plants in the absence of real measurements.
5. In order to provide more accurate information for environmental impact prediction and assessment, which is also the main direction of future research in this research, SACTI model calculation results are stored directly in ASCII code text data, which has no spatial relationship and cannot be used for secondary data analysis by GIS tools, such as spatial overlay with population or land use data.
6. The SACTI model is essentially a Gaussian plume model, which is a widely-used approach for estimating cooling tower plume dispersion. However, the SACTI model still has a number of limitations related to its Gaussian assumptions. For example, it assumes that air pollutant dispersion follows a symmetrical, bell-shaped curve from its source, which may not accurately represent real-world conditions. Moreover, the model assumes that air pollutant concentrations decrease exponentially from the source, which may not hold true for sources with non-uniform emissions or where local topography plays a substantial role in dispersion. Additionally, the SACTI model does not account for some of the more complex atmospheric processes that can impact air pollutant dispersion, such as turbulent mixing or local wind patterns. These limitations should be carefully considered when selecting and applying the SACTI model for environmental impact assessments. Thus, in future, we will use CFD model to carry out research on Cooling Tower plume dispersion.

## Data Availability

The datasets generated during and/or analysed during the current study are available from the corresponding author on reasonable request.

## Abbreviations

SACTI: Seasonal and annual cooling tower impact
PREP: Pre-processing module
MULT: Plume calculation module
TABLE: Table output module
PAGEPLOT: Printout module
CD144: Meteorological data formats used in the SACTI model
CFD: Computational fluid dynamics
